# An open chat with… Cecília Maria Arraiano

**DOI:** 10.1002/2211-5463.13604

**Published:** 2023-04-19

**Authors:** Ruzhica Bogeska, Cecília Maria Arraiano

**Affiliations:** ^1^ FEBS Open Bio Editorial Office Cambridge UK; ^2^ Instituto de Tecnologia Química e Biológica António Xavier Universidade Nova de Lisboa Oeiras Portugal

## Abstract

Professor Cecília Maria Arraiano directs a research group named ‘Control of Gene Expression’ at Instituto de Tecnologia Química e Biológica, Universidade NOVA de Lisboa, Oeiras, Portugal. She started her scientific journey at the University of Lisbon, where she graduated in Biology, before completing her PhD in Genetics as a Fulbright‐Hays Fellow at the University of Georgia, Athens, USA. After a postdoc in the USA, she returned to Lisbon to establish her own lab. She has authored close to 200 publications mainly in the field of RNA degradation mechanisms, with a focus on enzymes and RNA chaperones that mediate RNA decay in microorganisms. She has received several prizes and is an active member of prestigious organizations. Namely, she is an EMBO member, Fellow of the European Academy of Microbiology, Fellow of the American Academy of Microbiology, and member of the Portuguese Academy of Sciences. In addition, Prof Arraiano has chaired the FEBS Working Group on Women in Science from 2014 to 2022. In this fascinating interview, she discusses her research, her experience working in the USA and Portugal, and the importance of initiatives to support women in science.

## Graphical abstract

Professor Cecília Maria Arraiano directs a research group at Instituto de Tecnologia Química e Biológica, Universidade NOVA de Lisboa, Oeiras, Portugal. Her scientific journey began at the University of Lisbon, and she completed her PhD at the University of Georgia. Here, she discusses her research, her experience working in the USA and Portugal, and the importance of initiatives to support women in science.

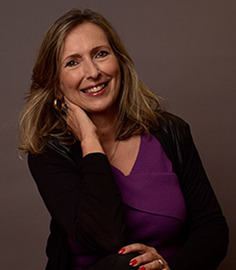

## For a nonspecialist audience, how would you describe the central theme of your research?

The central theme of my research is RNA, and how it controls the life of the cell. For a long time, DNA was the center of attention, but now, RNA has shown that it is much more than just a translator of the DNA information. The recent pandemic crisis due to the SARS‐CoV‐2 RNA virus has highlighted the importance of RNA studies. In a simplistic way, DNA can be said to be the architect that gives the genetic plan, but there are many types of RNA—'the engineers, the constructors, and the coordinators’—that can change the work in consideration of the environment, the stresses, and the ‘budget’, and the final work can be quite different from the initial DNA ‘sketch’.

## What events guided you to study the post‐transcriptional control of gene expression?

For many years, researchers focused on the transcription of RNA, its synthesis. However, in my PhD dissertation, I focused on the degradation of RNA and the ribonucleases, the enzymes that process, degrade, and control turnover of different types of RNA, which are essential molecules in the cell. In fact, the steady‐state levels of any type of RNA are a result of a balance between their synthesis and degradation. Many works have shown that post‐transcriptional control is a determinant factor in the maturation of RNAs, including the transfer and ribosomal RNAs, which determine the production of proteins. The RNases degrade the RNAs and control turnover of the RNAs adjusting them to the needs of the cell. Furthermore, they perform quality control of RNA by cleaving those that are abnormal.

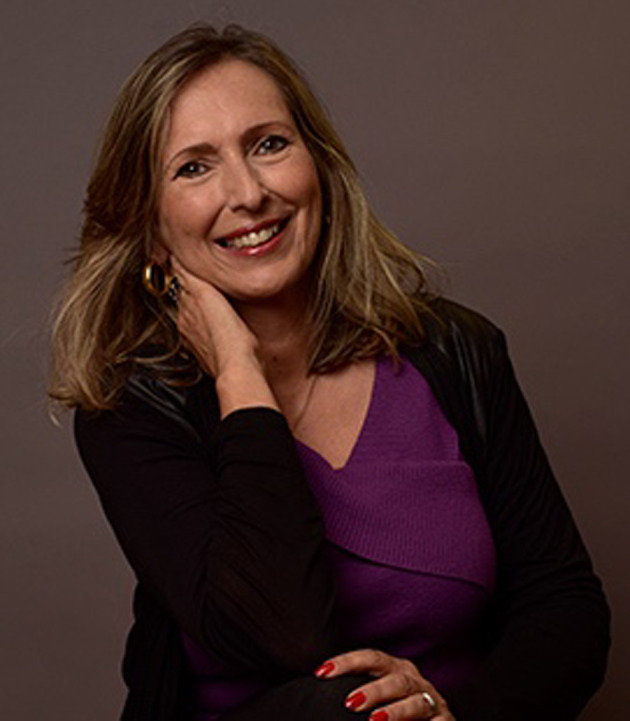

## In your field of research, which scientific or technological discovery do you consider to be the most important?

In my field of research, I believe that the discovery of noncoding RNAs in all kingdoms of life was fantastic and I am glad that I have also worked in this area and contributed with important discoveries. To give examples of the relevance of this scientific discovery, two Nobel prizes related to noncoding RNAs were awarded: in 2006 for RNA interference and in 2020 for CRISPr systems.

## You studied in the USA before establishing your own lab in Lisbon. Did you find that the research environment changed in Portugal during the years you were in the USA? What were the most striking changes?

When I arrived from the USA, the research environment in Portugal was much behind, but during the years, it has substantially improved and now we have top research institutes. I think that a great advance was due to the incentives given by a previous Science Minister—Prof Mariano Gago. However, in recent years, the Portuguese government has given little interest to science and has substantially reduced funding for scientific projects. I hope things rapidly improve….

## What challenges did you face when establishing your lab at Instituto de Tecnologia Química e Biológica, Universidade Nova de Lisboa, Oeiras, Portugal?

Many, many challenges…first the institute was not completely ready, so I had to help and give ideas in many areas different from mine, and start everything from scratch… But fortunately, the scientists that arrive now have a fully operational institute of international standards.

## What would you say are the pros and cons of pursuing a scientific career in the USA versus Portugal?

In the USA, the PhD program is 5 years, with 1 year dedicated to many graduate courses, which give us excellent preparation in the field. In Portugal, the PhD program is 4 years (at ITQB, they have 6 months of courses) and, in several other European countries, only 3 years. When applying for a postdoctoral fellowship, a longer PhD has more chances of resulting in good publications and therefore more possibilities for competitive positions. The cons are that when I arrived, I had to try to get integrated into European research by attending many conferences and workshops to meet people and make contacts.

## You are currently a member of EMBO. In your opinion, what are the major similarities and/or differences between EMBO and FEBS?

To be elected as an EMBO member (a lifetime position), you have to show that you have made recognized, international contributions in your scientific area. Consequently, an EMBO membership shows that you somewhat belong to an ‘elite’ of chosen European scientists. Regarding FEBS, you can become a FEBS member just by being a member of a national biochemical society, which belongs to FEBS. However, the FEBS Board of Trustees (Executive Board) has distinguished people who were elected by the FEBS Council. Both EMBO and FEBS promote science through workshops, fellowships, and all type of events, and I believe that both are essential for the progress of science in Europe and beyond.

## You were elected to chair the FEBS Working Group on Women in Science for three terms, from 2014 to 2022. What do you consider to be your top achievement during this time?

I believe that my top achievement was to give visibility to fantastic women in science as role models and to motivate young researchers to continue science, besides many gender‐related difficulties. During this time, I have met extraordinary people; I cannot forget an Italian girl who told me that she was about to quit, but after attending a FEBS Symposium on Gender Issues in Science, she changed her opinion and was going to continue in science!

## During your mandate, have you observed any improvements in terms of gender balance within and outside of FEBS?

I am glad that I have observed improvements within and outside FEBS, but….in certain countries, the freedom has receded by centuries and women are not free to pursue their education and to express themselves. It is dramatic!

## Would you recommend a more tailored approach of supporting women in science, rather than fulfilling quotas, for example, that would better facilitate the balance between personal and career choices? In your opinion, how could this be archived?

I am certainly in favor of a tailored approach of supporting women in science. However, when I was younger I thought that quotas were an embarrassment for smart women… but now that I am older, and with much more experience, I see that, for now, quotas are still a must: for instance, I have evaluated European projects in which two men who were PIs were putting forward their secretaries as Scientific PIs to pretend that the project had gender balance. And it is shocking that I have realized that in some Horizon Europe projects, gender balance only refers to the animals used in the experiments, not to the scientists in the consortium…

## Could you list prizes or programs aimed at supporting women, diversity, and equality in science that you consider the most helpful?

I think that the FEBS/EMBO Women in Science Award and the L'Oréal International Prize are important examples that give great visibility to women as role models in science. L'Oréal together with UNESCO and National Foundations also give annual prizes, to support research of women under 35.

## Both Florence Bell and Rosalind Franklin have been referred to as ‘The dark ladies of DNA’ due to the insufficient acknowledgment of their major contributions to science. Although there has been significant progress in acknowledging scientists for their discoveries regardless of their gender or personal convictions from the mid‐20th century to the present, are you aware of any form of discrimination that is currently ongoing and is keeping researchers figuratively working ‘in darkness’?

Certainly, there are still many examples of women working in the darkness, but I am also sure that there many examples of men working in the darkness for many different reasons: due to difficulties of hierarchy, the society/country where they live, the topic in which they work, and even financial struggles to survive.

It just occurred to me that I heard Katalin Karikó talking in a seminar held by the Rosalind Franklin Society. She told us that she was working with human coronaviruses—the cause of the common cold. She had huge difficulties in finding financial support because nobody cared about her research and her search for an RNA vaccine for coronavirus. Then, the COVID‐19 pandemic arrived and due to her research ‘in the dark’, she has now finally been recognized for her major contribution to an RNA vaccine against SARS‐Cov‐2.

## Do you have a personal motto you would like to share?

I believe that you have to really love science in order to be persistent and overcome the daily obstacles in your research. If you like science but you are not in love, then my advice is to pursue something related to science, but without immersing yourself into it. It is good to make the choice that makes you happy!

